# Taxonogenomics description of *Parabacteroides timonensis* sp. nov. isolated from a human stool sample

**DOI:** 10.1002/mbo3.702

**Published:** 2018-10-11

**Authors:** Melhem Bilen, Maxime Descartes Mbogning Fonkou, Saber Khelaifia, Enora Tomei, Frédéric Cadoret, Ziad Daoud, Nicholas Armstrong, Fadi Bittar, Pierre‐Edouard Fournier, Didier Raoult, Gregory Dubourg

**Affiliations:** ^1^ Aix Marseille University IRD AP‐HM MEPHI IHU Méditerranée‐Infection Marseille France; ^2^ Aix Marseille University IRD, AP‐HM, SSA VITROME IHU Méditerranée‐Infection Marseille France; ^3^ Assistance Publique Hôpitaux de Marseille (AP‐HM) IHU Méditerranée‐Infection Marseille France; ^4^ Clinical Microbiology Department Faculty of Medicine and Medical sciences University of Balamand Amioun Lebanon; ^5^ Special Infectious Agents Unit King Fahd Medical Research Center King Abdulaziz University Jeddah Saudi Arabia

**Keywords:** culturomics, human, microbiome, new species, *Parabacteroides timonensis*

## Abstract

Intensive efforts have been made to describe the human microbiome and its involvement in health and disease. Culturomics has been recently adapted to target formerly uncultured bacteria and other unclassified bacterial species. This approach enabled us to isolate in the current study a new bacterial species, *Parabacteroides timonensis* strain Marseille‐P3236^T^, from a stool sample of a healthy 39‐year‐old pygmy male. This strain, is an anaerobic, gram‐negative, nonspore‐forming motile rod. Its genome is made up of 6,483,434 bp with 43.41% G+C content, 5046 protein‐encoding genes, and 84 RNA genes. We herein provide the full description of *Parabacteroides timonensis* strain Marseille‐P3236^T^ through the taxonogenomic approach.

## 1 INTRODUCTION

The gut microbiota is well‐known for its microbial diversity and its role in health as well as in diseases. Even though scientific technologies have been greatly developed over the past years and have drastically facilitated the description of the gut microbiota, it still remains a challenging task (Turnbaugh et al., 2007) as the massive data generated over the last decade do not yet allow the clear depiction of the gut microbiota composition (Lagier et al., 2012). Nevertheless, the fact that 1 g of human stool might contain up to 10^12^ bacteria drives us to pursue our efforts in describing the human gut microbiota for which only around 2,776 species have been reported (Bilen et al., 2018; Hugon et al., 2015). Consequently, our laboratory has developed a new approach called culturomics which aims to isolate previously uncultured bacteria using sophisticated culture methods (Lagier et al., 2012). In doing so, culturomics has expanded our capabilities in human gut microbiota description and therefore lead to the isolation of a significant number of new genera and species (Lagier et al., 2016). The process begins with the cultivation of stool samples under varying conditions and bacterial growth is assessed over 30 days. At this point, Matrix‐assisted laser desorption/ionization time‐of‐flight mass spectrometry (MALDI‐TOF MS) is primarily used for colony identification and 16S rRNA sequencing is adapted in case of MALDI‐TOF MS's identification failure. Subsequently, unidentified species are subjected to taxonogenomics description (Fournier & Drancourt, 2015). The genome of the concerned species is then sequenced for a genomic description, followed by a phenotypic and biochemical analysis (Fournier & Drancourt, 2015; Fournier, Lagier, Dubourg, & Raoult, 2015; Lagier et al., 2012). By adapting this procedure, we isolated a new species known as *Parabacteroides timonensis* (*P. timonensis*), a member of the *Parabacteroides* genus known to be gram‐negative, obligate anaerobic, nonmotile, rod‐shaped, and nonspore‐forming (Sakamoto & Benno, 2006). To date, eight *Parabacteroides* species have been isolated, out of which six were isolated from the human gut (www.bacterio.net). We demonstrate here the description of *P. timonensis* strain Marseille‐P3236^T^ (=Culture Collection University Gothenburg (CCUG) 71183, =Collection de Souches de l'Unité des Rickettsies (CSUR) P3236) using the taxonogenomic approach.

## 2 MATERIALS AND METHODS

### 2.1 Ethics and sample collection

Before stool sample collection in Congo, the sample's donor has signed an informed consent. The donor is a healthy 39‐year‐old pygmy male and the collected stool samples were stored at −80°C for further analysis. In addition, an approval from the ethic committee of the Institut Fédératif de Recherche IFR48 (Marseille, France) carrying the number 09‐022 was obtained before launching the study.

### 2.2 Strain isolation

A loop of stool sample was diluted in phosphate‐buffered saline (Life Technologies, Carlsbad, CA, USA) prior to incubation in a blood culture bottle (BD BACTEC^®^, Plus Anaerobic/F Media, Le Pont de Claix, France), supplemented with 5% sheep blood and 5% filtered rumen, at 37°C under anaerobic conditions. Bacterial growth and isolation was done by subculturing samples after 5 days on 5% sheep's blood–enriched Columbia agar solid medium (bioMérieux, Marcy l'Etoile, France).

### 2.3 Colony identification

Isolated bacterial colonies identification trials were done first by using MALDI‐TOF MS analysis as previously described (Elsawi et al., 2017). In case of MALDI‐TOF MS identification failure, complete 16S rRNA sequencing was performed for further analysis with the same protocol used in our previous studies (Elsawi et al., 2017). Complete 16S rRNA nucleotide sequence are assembled and manipulated using CodonCode Aligner software (http://www.codoncode.com) and blasted in the online PubMed National Center for Biotechnology Information (NCBI) database for phylogenetic analysis. According to Kim, Oh, Park, & Chun (2014), a threshold of 98.65% 16S rRNA gene sequence similarity was used to classify a new species, whereas a threshold of 95% 16S rRNA gene sequence was used for new genus classification. Generated mass spectrum of the concerned species was added to our custom database and its 16S rRNA gene sequence was submitted to EMBL‐EBI database.

### 2.4 Growth conditions

In order to determine the optimal growth environment, strain Marseille‐P3236^T^ was cultured using different conditions such as temperature, pH, atmosphere, and salinity. To begin with, this strain was cultured under anaerobic, aerobic, and microaerophilic conditions on 5% sheep's blood–enriched Colombia agar (bioMérieux) at 28, 45, 37, and 55°C. GENbag anaer and GENbag microaer systems (bioMérieux) were used for anaerobic and microaerophilic environment establishment, respectively. Furthermore, salt and acidity tolerance were evaluated using concentration of 0%, 5%, 15%, and 45% NaCl and pH values of 6, 6.5, 7, and 8.5.

### 2.5 Morphological and biochemical assays

Biochemical characteristics of Marseille‐P3236^T^ strain were determined using different API galleries (20A, ZYM, and 50CH, bioMérieux) according to the manufacturer's instructions. Not to mention, sporulation ability was tested by culturing this strain after exposing a bacterial suspension to a thermic shock of 80°C for 10 min. Strain Marseille‐P3236^T^ morphology was determined as previously described (Elsawi et al., 2017). Additionally, DM1000 photonic microscope (Leica Microsystems, Nanterre, France) was used to observe the motility of strain Marseille‐P3236^T^ from a fresh culture with a 100× objective lens. Cellular fatty acid methyl ester (FAME) analysis was performed using around 43 mg of bacterial biomass per tube as formerly described (Elsawi et al., 2017).

### 2.6 Antibiotic susceptibility

Antibiotics susceptibility testing (AST) of Marseille P‐3236^T^ strain was evaluated by performing minimum inhibitory concentrations (MICs) using the *E*‐test strip method (Biomérieux) on Columbia agar + 5% sheep blood (Biomérieux) with the following agents: rifampicin, imipenem, amikacin, cefotaxime, benzylpenicillin, vancomycin, erythromycin, amoxicillin, ceftriaxone, minocycline, metronidazole, teicoplanin, and daptomycin.

### 2.7 Genome sequencing and analysis

Genomic DNA (gDNA) of Marseille‐P3236^T^ strain was extracted as previously described with 50 μL being eluted (Elsawi et al., 2017). Quantification was done using the Qubit assay with the high sensitivity kit (Life technologies, Carlsbad, CA, USA) and determined to be 134 ng/μl.

gDNA sequencing, library preparation, fragmentation, and tagmentation were performed as previously described with an optimal DNA fragment size of 8.675 kb (Lagier et al., 2016). Circularization was done using 511.4 ng of tagmented fragments with no size selection. The circularized DNA was mechanically sheared to small fragments with optima at 1059 bp on the Covaris device S2 in T6 tubes (Covaris, Woburn, MA, USA). Library profile visualization was done on a High Sensitivity Bioanalyzer LabChip (Agilent Technologies Inc, Santa Clara, CA, USA) and the final library concentration was determined at 27.8 nmol/L. After that, libraries were normalized at 2 nM and pooled. Dilution at 15 pM was done after a denaturation step and pooled libraries were loaded in the sequencer for automated cluster generation and a single sequencing run of 39 h in a 2 × 151‐bp was performed. Total information of 5.1 Gb were generated from a 542 K/mm^2^ cluster density with a quality control filters threshold of 95.7% (10,171,000 passing filter paired reads). Within this run, index representation for Marseille‐P3236^T^ strain was determined to 7.69%. The 782,587 paired reads were trimmed and then assembled. Genome assembly, annotation, and comparison were done using the same pipeline and tools as previously described (Elsawi et al., 2017).

## 3 RESULTS AND DISCUSSION

### 3.1 Strain identification and phylogenetic analysis

**Figure 1:**
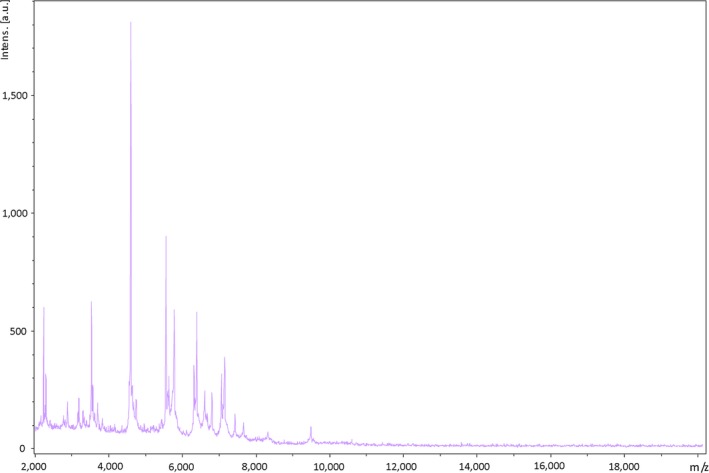
Reference mass spectrum representing *Parabacteroides timonensis* strain Marseille‐P3236^T^

**Figure 2:**
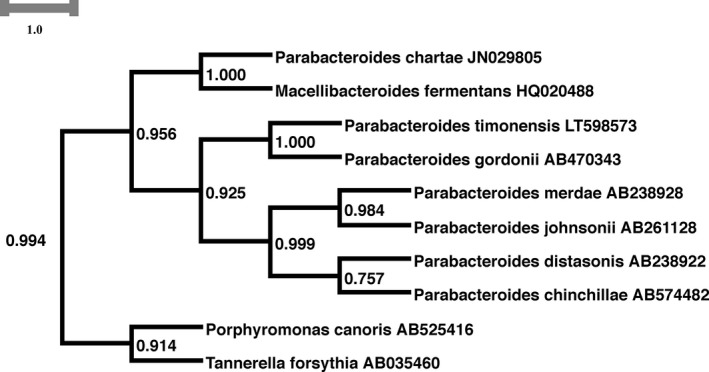
Phylogenetic subtree highlighting the position of *Parabacteroides timonensis* strain Marseille‐P3236^T^ relative to other close species

**Figure 3:**
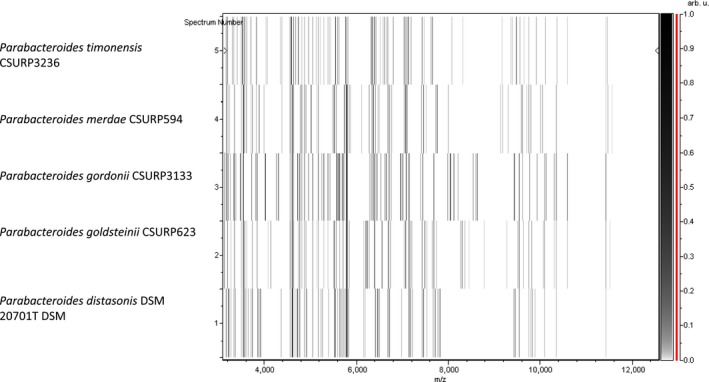
Gel view comparing mass the mass spectrum of *Parabacteroides timonensis* strain Marseille‐P3236^T^ to other species. The gel view displays the raw spectra of loaded spectrum files arranged in a pseudo‐gel like look. The *x*‐axis records the m/z value. The left *y*‐axis displays the running spectrum number originating from subsequent spectra loading. The peak intensity is expressed by a Gray scale scheme code. The right *y*‐axis indicates the relation between the color of a peak and its intensity, in arbitrary units. Displayed species are indicated on the left

The identification of P3236^T^ strain using MALDI‐TOF MS failed due to the absence of its mass spectrum in the current databases. However, a typical spectrum was added to our custom database (Figure 1) after performing a complete 16s rRNA gene sequencing. Strain Marseille‐P3236^T^ exhibited a 97.05% sequence similarity with *Parabacteroides gordonii* strain MS‐1 (NR_112835.1), the phylogenetically closest species with standing nomenclature (Figure 2). Accordingly, with more than 1.3% sequence divergence from its phylogenetically closest species with standing in nomenclature, we propose the classification of Marseille‐P3236^T^ strain as a new species within the *Parabacteroides* genus. The protein spectrum of Marseille‐P3236^T^ strain was compared with those of other close species under the same family. This comparison revealed its uniqueness with specific and different peaks compared to others (Figure 3).

### 3.2 Phenotypic and biochemical characterization

**Figure 4:**
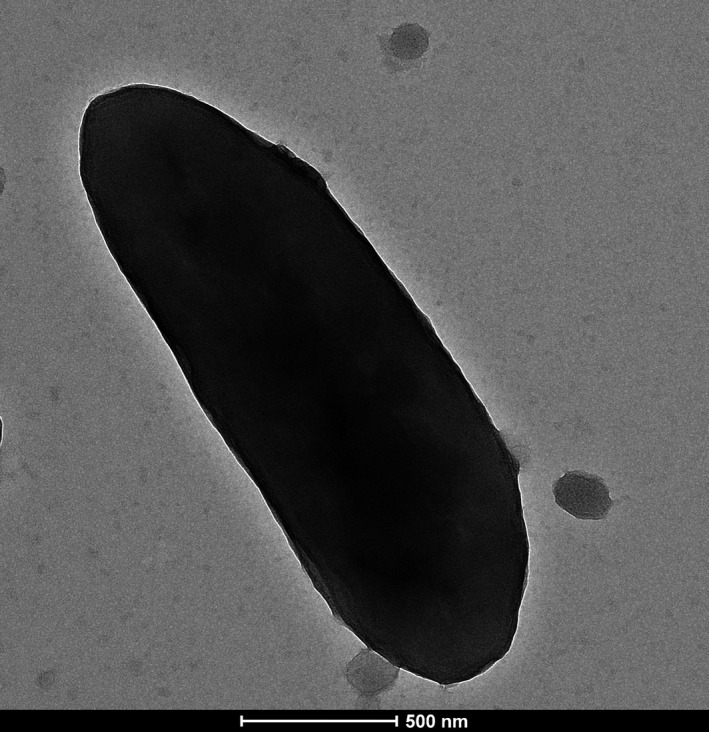
Electron micrographs of *Parabacteroides timonensis* strain Marseille‐P3236^T^ using a Tecnai G20, at an operating voltage of 200 keV. Scale bar = 200 nm

Strain Marseille‐P3236^T^ is a gram‐negative rod, motile, unable to sporulate, and grows anaerobically between 25 and 42°C but optimally at 37° (Table 1). It is able to endure a range of pH between 6 and 8.5 and can sustain only a 5% salinity concentration. Strain Marseille‐P3236^T^ is catalase positive, oxidase negative, and can be seen as smooth colonies with a diameter of 0.9–1 mm. Under electron microscopy, each bacterial cell has an average length of 1.4–2.7 μm and an average diameter of 0.5 μm (Figure 4).

**Table 1:** Classification and general features of *Parabacteroides timonensis* strain Marseille‐P3236^T^

Property	Term
Current classification	Domain: *Bacteria*
	Phylum: *Bacteroidetes*
	Class: *Bacteroidia*
	Order: *Bacteroidales*
	Family: *Porphyromonadaceae*
	Genus: *Parabacteroides*
	Species: *Parabacteroides timonensis*
	Type strain: P3236
Gram strain	Negative
Cell shape	Rod
Motility	Motile
Sporulation	Negative
Temperature range	30‐42°C
Optimum temperature	37°C

MICs of strain Marseille‐P3236^T^ for various antimicrobial agents were as follows: rifampicin (8 μg/ml), Imipenem (8 μg/ml), amikacin (>256 μg/ml), cefotaxime (6 μg/ml), benzylpenicillin (16 μg/ml), vancomycin (4 μg/ml), erythromycin (0.016 μg/ml), amoxicillin (1.5 μg/ml), ceftriaxone (4 μg/ml), minocycline (0.25 μg/ml), metronidazole (>256 μg/ml), teicoplanin (2 μg/ml), and daptomycin (12 μg/ml).

The main biochemical features obtained by API tests are summarized in Table 2. Additionally, the general features of strain Marseille‐P3236^T^ have been listed in Table 3.

**Table 2:** Main biochemical features of strain *Parabacteroides timonensis* strain Marseille‐P3236^T^ obtained by API tests (20A, 50CH, and ZYM)

Test	Results	Test	Results	Test	Results
Alkaline phosphatase	+	Fermentation (d‐glucose)	+	Fermentation (d‐turanose)	+
Esterase (C4)	+	Fermentation (d‐fructose)	+	Fermentation (d‐lyxose)	−
Esterase Lipase (C8)	+	Fermentation (d‐mannose)	+	Fermentation (d‐tagatose)	+
Lipase (C14)	−	Fermentation (l‐sorbose)	−	Fermentation (d‐fucose)	−
Leucine arylamidase	+	Fermentation (l‐rhamnose)	+	Fermentation (l‐fucose)	−
Valine arylamidase	+	Fermentation (d‐ulcitol)	−	Fermentation (d‐arabitol)	−
Cystine arylamidase	+	Fermentation (Inositol)	−	Fermentation (l‐arabitol)	−
Trypsin	−	Fermentation (d‐mannitol)	+	Fermentation (potassium gluconate)	+
α‐chymotrypsin	−	Fermentation (d‐sorbitol)	+	Fermentation (potassium 2‐Ketogluconate)	−
Acid phosphatase	+	Fermentation (Methyl‐αd‐mannopyranoside)	+	Fermentation (potassium 5‐Ketogluconate)	−
Naphthol‐AS‐BI‐phosphohydrolase	+	(Fermentation (Methyl‐αd‐glucosamine))	+	Indole formation	−
α‐galactosidase	+	Fermentation (*n*‐acetylglucosamine)	+	Urease	−
β‐galactosidase	+	Fermentation (Amygdaline)	+	Acidification (Glucose)	+
β‐glucuronidase	+	Fermentation (Arbutin)	+	Acidification (Mannitol)	−
α‐glucosidase	+	Fermentation (Esculin ferric citrate)	+	Acidification (Lactose)	+
β‐glucosidase	+	Fermentation (Salicin)	+	Acidification (Saccharose)	+
*N‐*acetyl‐β‐glucosaminidase	+	Fermentation (d‐cellobiose)	+	Acidification (Maltose)	+
α‐mannosidase	−	Fermentation (d‐Maltose)	+	Acidification (Salicin)	−
α‐fucosidase	−	Fermentation (d‐lactose)	+	Acidification (Xylose)	+
Fermentation (Glycerol)	−	Fermentation (d‐melibiose)	+	Acidification (Arabinose)	+
Fermentation (Erythritol)	+	Fermentation (d‐saccharose/sucrose)	+	Hydrolysis (protease) (Gelatin)	−
Fermentation (d‐arabinose)	+	Fermentation (d‐trehalose)	+	Hydrolysis β‐ glucosidase (Esculin)	−
Fermentation (l‐arabinose)	+	Fermentation (Inuline)	+	Acidification (Glycerol)	−
Fermentation (d‐ribose)	+	Fermentation (d‐melezitose)	+	Acidification (Cellobiose)	−
Fermentation (d‐xylose)	+	Fermentation (d‐raffinose)	+	Acidification (Mannose)	+
Fermentation (l‐xylose)	−	Fermentation (Starch)	+	Acidification (Melezitose)	−
Fermentation (d‐adonitol)	−	Fermentation (Glycogen)	−	Acidification (Raffinose)	+
Fermentation (Methyl‐β‐d‐xylopyranoside)	−	Fermentation (xylitol)	−	Acidification (Sorbitol)	−
Fermentation (d‐galactose)	+	Fermentation (Gentiobiose)	+	Acidification (Rhamnose)	+
				Acidification (Trehalose)	+

**Table 3:** General characteristics of *Parabacteroides timonensis* strain Marseille‐P3236^T^

Properties	*Parabacteroides timonensis*
Cell length (μm)	1.4‐2.7
Oxygen requirement	Strictly anaerobic
Gram stain	Negative
Salt requirement	−
Motility	+
Endospore formation	−
Indole	−
Production of	
Alkaline phosphatase	+
Catalase	+
Oxidase	−
Urease	−
β‐galactosidase	+
*n‐*acetyl‐glucosamine	+
Acid from	
l‐Arabinose	+
d‐Ribose	+
d‐Mannose	+
d‐Mannitol	+
d‐glucose	+
d‐fructose	+
d‐maltose	+
d‐lactose	+
G+C content (mol%)	43.41
Habitat	Human gut

The major fatty acid found in this strain was 12‐methyl‐tetradecanoic acid (46%). Several specific 3‐hydroxy branched structures were described. Minor amounts of unsaturated, branched, and other saturated fatty acids were also detected (Table 4).

**Table 4:** Cellular fatty acids composition of *Parabacteroides timonensis* strain Marseille‐P3236^T^

Fatty acids	Name	Mean relative % (a)
15:0 anteiso	12‐methyl‐tetradecanoic acid	46.0 ± 0.5
15:0	Pentadecanoic acid	8.6 ± 0.4
18:1n9	9‐Octadecenoic acid	8.4 ± 0.4
17:0 3‐OH iso	3‐hydroxy‐15‐methyl‐Hexadecanoic acid	7.6 ± 0.4
16:0	Hexadecanoic acid	6.1 ± 0.1
18:2n6	9,12‐Octadecadienoic acid	5.8 ± 0.5
15:0 iso	13‐methyl‐tetradecanoic acid	4.7 ± 0.1
16:0 3‐OH	3‐hydroxy‐Hexadecanoic acid	3.9 ± 0.3
17:0 3‐OH anteiso	3‐hydroxy‐14‐methyl‐Hexadecanoic acid	1.4 ± 0.6
14:0 iso	12‐methyl‐Tridecanoic acid	1.2 ± 0.1
5:0 iso	3‐methyl‐butanoic acid	1.0 ± 0.1
14:0	Tetradecanoic acid	1.0 ± 0.1
18:0	Octadecanoic acid	TR
16:1n7	9‐Hexadecenoic acid	TR
15:0 3‐OH	3‐hydroxy‐Pentadecanoic acid	TR
17:0 3‐OH	3‐hydroxy‐Heptadecanoic acid	TR
17:1n7	10‐Heptadecenoic acid	TR
20:4n6	5,8,11,14‐Eicosatetraenoic acid	TR
13:0 iso	11‐methyl‐Dodecanoic acid	TR
18:1n7	11‐Octadecenoic acid	TR
16:0 3‐OH iso	3‐hydroxy‐14‐methyl‐Pentadecanoic acid	TR
16:0 9,10‐methylene	2‐hexyl‐Cyclopropaneoctanoic acid	TR
13:0 anteiso	10‐methyl‐Dodecanoic acid	TR
17:0	Heptadecanoic acid	TR
13:0	Tridecanoic acid	TR

Mean peak area percentage; TR = trace amounts <1%.

### 3.3 Genome properties

**Figure 5:**
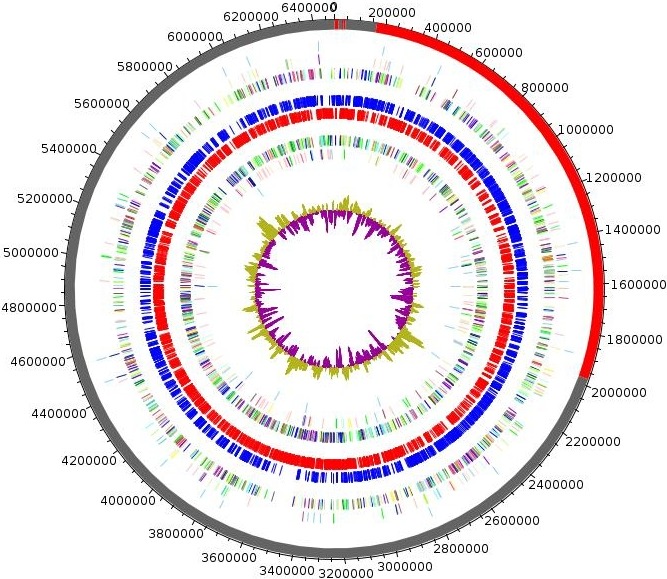
Graphical circular map of the genome of *Parabacteroides timonensis* strain Marseille‐P3236^T^. From outside to the center: Contigs (red/gray), COG category of genes on the forward strand (three circles), genes on forward strand (blue circle), genes on the reverse strand (red circle), COG category on the reverse strand (three circles), G+C content

Strain Marseille‐P3236^T^ had a genome of 6,483,434 bp long with 43.41 mol% of G+C content. It is composed of eight scaffolds (composed of 13 contigs). Of the 5,130 predicted genes, 5,046 were protein‐coding genes and 84 were RNAs (6 genes are 5S rRNA, 1 gene is 16S rRNA, 5 genes are 23S rRNA, and 72 genes are tRNA genes). A total of 3,253 genes (64.47%) were assigned as putative function (by cogs or by NR blast) and 202 genes were identified as ORFans (4%). The remaining genes were annotated as hypothetical proteins (1,483 genes; 29.39%) (Table 5). The representations of strain Marseille‐P3236^T’^ genome and its genes repartition into COG functional categories were done in Figure 5 and Table 6, respectively.

**Table 5:** General genomic characteristics of strain Marseille‐P3236^T^

	Number	Percent^a^
Size (bp)	6,483,434	100
Number of G+C	2,813,492	43.41
Number total of genes	5,130	100
Number total of protein genes	5,046	98.36
Number total of RNA genes	84	1.64
Number total of tRNA genes	72	1.40
Number total of RNA (5S, 16S, 23S) genes	12	0.23
Coding sequence size	5,884,794	90.77
Coding sequence gene protein size	5,858,682	90.36
Coding sequence tRNA gene size	5,555	0.09
Coding sequence (5S, 16S, 23S) gene size	20,557	0.32
Number of protein‐coding gene	5,046	100
Number of protein associated to COGs	2,513	49.80
Number of protein associated to orfan	202	4.00
Number of protein with peptide sigNAl	1,641	32.52
Number of gene associated to resistance genes	0	0
Number of gene associated to PKS or NRPS	11	0.22
Number of genes associated to virulence	705	13.97

a The total is based on either the size of the genome in base pairs or the total number of protein‐coding genes in the annotated genome.

**Table 6:** Number of genes associated with the 25 general COG functional categories

Code	Value	% of total	Description
[J]	194	3.8446293	Translation
[A]	0	0	RNA processing and modification
[K]	194	3.8446293	Transcription
[L]	165	3.2699168	Replication, recombination and repair
[B]	0	0	Chromatin structure and dynamics
[D]	25	0.49544194	Cell cycle control, mitosis, and meiosis
[Y]	0	0	Nuclear structure
[V]	128	2.5366626	Defense mechanisms
[T]	183	3.6266348	Signal transduction mechanisms
[M]	233	4.617519	Cell wall/membrane biogenesis
[N]	22	0.43598887	Cell motility
[Z]	1	0.019817676	Cytoskeleton
[W]	0	0	Extracellular structures
[U]	37	0.7332541	Intracellular trafficking and secretion
[O]	96	1.902497	Posttranslational modification, protein turnover, chaperones
[X]	25	0.49544194	Mobilome: prophages, transposons
[C]	142	2.8141103	Energy production and conversion
[G]	248	4.914784	Carbohydrate transport and metabolism
[E]	180	3.5671818	Amino acid transport and metabolism
[F]	67	1.3277843	Nucleotide transport and metabolism
[H]	131	2.5961156	Coenzyme transport and metabolism
[I]	83	1.6448673	Lipid transport and metabolism
[P]	297	5.8858504	Inorganic ion transport and metabolism
[Q]	36	0.7134364	Secondary metabolites biosynthesis, transport and catabolism
[R]	229	4.538248	General function prediction only
[S]	108	2.140309	Function unknown
_	2,533	50.19818	Not in COGs

### 3.4 Comparison of genome properties

The draft genome sequence of Marseille‐P3236^T^ strain was compared to those of *Parabacteroides gordonii* (*P. gordonii*) (AUAE00000000), *Parabacteroides distasonis* (*P. distasonis*) (JNHP00000000), *Porphyromonas canoris* (*P. canoris*) (JQZV00000000), *Parabacteroides johnsonii* (*P. johnsonii*) (ABYH00000000), *Parabacteroides merdae* (*P. merdae*) (AJPU00000000), and *Tannerella forsythia* (*T. forsythia*) (CP003191).

Genome of Marseille‐P3236^T^ strain was smaller than those of *P. gordonii* (6,483 and 6,677 MB, respectively), but was nevertheless larger than those of *P. distasonis*,* P. canoris*,* P. johnsonii*,* P. merdae*, and *T. forsythia* (5,316, 2,203, 4,787, 4,459, and 3,282 MB, respectively). The G+C content of Marseille‐P3236^T^ strain was smaller than those of *P. distasonis*,* P. canoris*,* P. johnsonii*,* P. merdae*,* T. forsythia*, and *P. gordonii* (43.41%, 44.85%, 44.75%, 45.2%, 45.25%, 47.14%, and 44.43%, respectively). The gene content of Marseille‐P3236^T^ strain was smaller than those of *P. gordonii* (5,046 and 5,326, respectively), but was larger than those of *P. distasonis*,* P. canoris*,* P. johnsonii*,* P. merdae*, and *T. forsythia* (4,800, 1,612, 4,515, 3,703, and 2,492, respectively).

**Figure 6:**
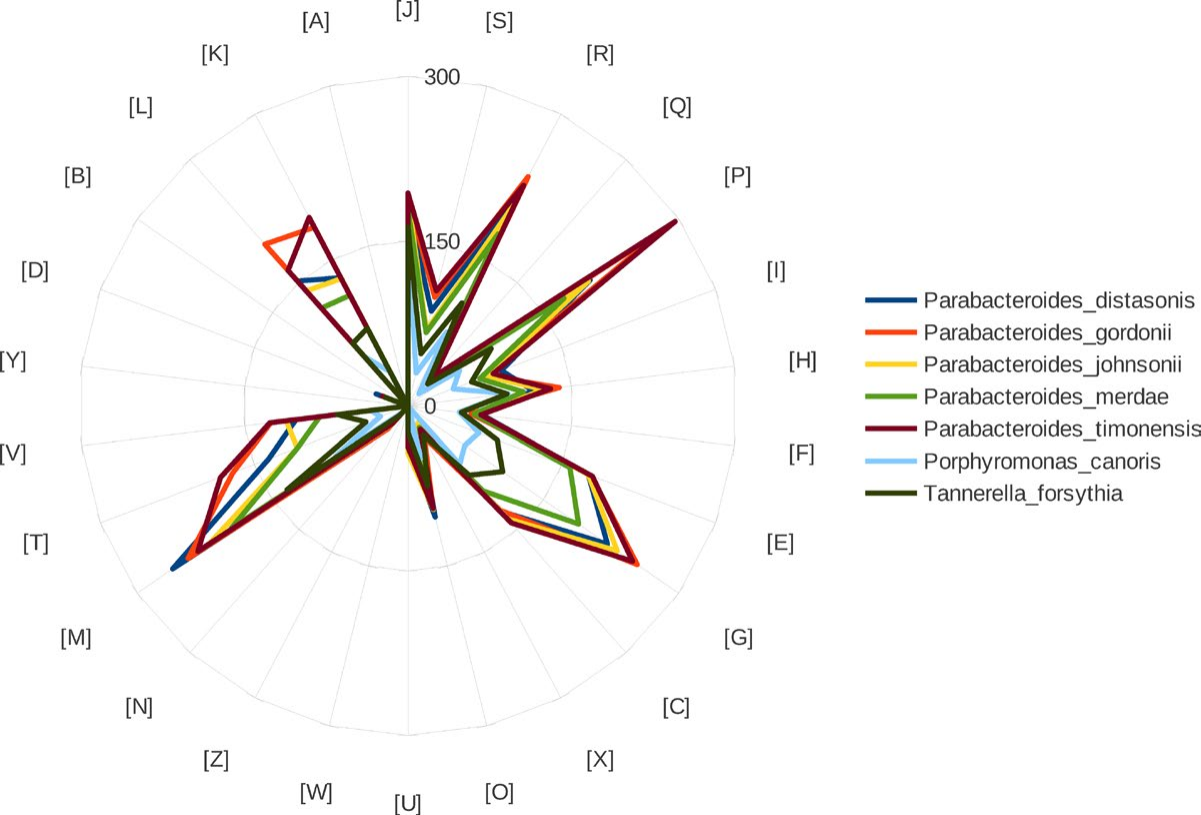
Distribution of functional classes of predicted genes according to the clusters of orthologous groups of proteins for *Parabacteroides timonensis* strain Marseille‐P3236^T^

Distribution of functional classes of predicted genes according to the COGs database regarding strain Marseille‐P3236^T^ is presented in Figure 6. The distribution was similar in all the studied genomes. Strain Marseille‐P3236^T^ shared the highest number of orthologous proteins with *P. gordonii* (2,614 with 84.62% similarity at the nucleotide level, Table 7).

**Table 7:** The numbers of orthologous proteins shared between strain Marseille‐P3236^T^ genomes and others closely related species genomes (upper right), average percentage similarity of nucleotides of shared orthologous proteins between genomes (lower left) and numbers of proteins per genome (bold)

	TF	PM	PC	PT	PJ	PG	PD
TF	**2,492**	1,287	822	1,388	1,207	1,379	1,248
PM	59.79	**3,703**	905	2,279	2,147	2,224	1,991
PC	56.21	55.97	**1,612**	935	860	922	871
PT	58.35	61.94	56.9	**5,046**	2,222	2,614	2,237
PJ	59.7	76.79	56.11	61.54	**4,515**	2,176	1,943
PG	58.45	62.12	56.94	84.62	61.71	**5,326**	2,216
PD	59.15	64.48	55.61	61.98	63.46	62	**4,800**

*Note*. TF, *Tannerella forsythia*; PM, *Parabacteroides merdae*; PC, *PorphyromoNAs canoris*; PT, *Parabacteroides timonensis*; PJ, *Parabacteroides johnsonii*; PG, *Parabacteroides gordonii*; PD, *Parabacteroides distasonis*.

As for digital DNA‐DNA hybridization (dDDH) values between strain Marseille‐P3236^T^ and its phylogenetic closest species, it was 21.6 [19.3–24], 31 [28.6–33.5], 21.5 [19.2–23.9], 28 [25.7–30.5], and 23.5 [21.2–25.9] with *P. johnsonii*,* P. canoris*,* P. distasonis*,* P. gordonii*, and *T. forsythia,* respectively. In fact, 70% dDDH value is considered as a threshold to define a new bacterial species (Tindall, Rosselló‐Móra, Busse, Ludwig, & Kämpfer, 2010; Wayne et al., 1987). According to our results, strain Marseille‐P3236^T^ shared with all its phylogenetically closest species with standing in nomenclature dDDH value of less than 70% and thus confirming it as a new species (Table 8).

**Table 8:** Pairwise comparison of *Parabacteroides timonensis* strain Marseille‐P3236^T^with other species using GGDC, formula 2 (DDH estimates based on identities/HSP length)^a^ upper right

	PT	PJ	PC	PD	PG	TF
PT	100%	21.6 [19.3–24%]	31 [28.6–33.5%]	21.5 [19.2–23.9%]	28 [25.7–30.5%]	23.5 [21.2–25.9%]
PJ		100%	30.8 [28.4–33.3%]	22.5 [20.2–24.9%]	21.3 [19.1–23.8%]	28.8 [26.4–31.3%]
PC			100%	28 [25.7–30.5%]	33.3 [25.7–30.5%]	32.9 [25.7–30.5%]
PD				100%	20.5 [18.3–23%]	23.2 [20.9–25.6%]
PG					100%	17.1 [15–19.5%]
TF						100%

*Note*. TF, *Tannerella forsythia*; PM, *Parabacteroides merdae*; PC, *PorphyromoNAs canoris*; PT, *Parabacteroides timonensis*; PJ, *Parabacteroides johnsonii*; PG, *Parabacteroides gordonii*; PD, *Parabacteroides distasonis*.

a The confidence intervals indicate the inherent uncertainty in estimating DDH values from intergenomic distances based on models derived from empirical test data sets.

## 4 DISCUSSION

The human gut microbiota has been extensively studied by the scientific community and it has already been correlated with several health conditions such as obesity (Million et al., 2016), gastrointestinal diseases (Guinane & Cotter, 2013), or nonalcoholic fatty acid liver disease (Abu‐Shanab & Quigley, 2010). Profiling the bacterial content and its ratio in the human gut have led to the development of several therapeutic strategies such as probiotics, and also therapeutic improvements such as the case of CTLA‐4‐based cancer immunotherapy (Vétizou et al., 2015). Hence, describing the human gut microbiota without neglecting a group of its population is essential. Culturomics was developed for the purpose of isolating previously uncultured organisms along with attempting to correlate its sequences to operational taxonomic units (Lagier et al., 2012, 2016). This work adds on the previously performed descriptive studies on the human gut microbiota via culturomics (Lagier et al., 2016) by isolating a new bacterial species belonging to the *Parabacteroides* genus (Sakamoto & Benno, 2006). However, *Parabacteroides* has been previously isolated from clinical cases (Kierzkowska et al., 2017) and was the causative agent of bacteremia (Awadel‐Kariem, Patel, Kapoor, Brazier, & Goldstein, 2010). This fact renders the isolation and description of a new *Parbacteroides* species important as it can now be more easily identified if isolated in the future during a clinical case and thus facilitating medical diagnoses.

## 5 CONCLUSION

In conclusion, describing the human microbiota remains a challenging task requiring intensive efforts. Even though sequencing approaches proved to be efficient in this field, culturomics reemphasized the importance of culture in deciphering the dark matter of the human microbiome by shedding the light on previously unidentified and uncultured species (Lagier et al., 2016). Herein, we report the isolation of a new bacterial species from the human gut, *Parabacteroides timonensis* strain P3236^T^ which represents the ninth *Parabacteroides* species and the seventh found in the human gut.

### 5.1 Description of *Parabacteroides timonensis* sp. nov

*Parabacteroides timonensis* (ti.mo.nen′sis. N.L. masc. adj. timonensis pertaining to La Timone, Marseilles’ hospital name, France, where the strain was isolated).

*P. timonensis* is motile, gram‐negative rod, catalase positive, oxidase negative, nonspore‐forming, strictly anaerobic, and has a cell length ranging between 1.4 and 2.7 μm. Colonies of *P. timonensis* are smooth and exhibit a diameter of 0.8–1 mm. The optimal growth temperature of *P. timonensis* is 37°C.

Positive reactions are observed with alkaline phosphatase, α‐glucosidase, esterase lipase C8, valine arylamidase, acid phosphatase, cystine arylamidase, α –galactosidase, naphthol‐AS‐BI‐phosphohydrolase, β‐galactosidase, leucine arylamidase, β‐glucuronidase, β‐glucosidase, esterase C4, *N*‐acetyl‐β‐glucosaminidase but negative with lipase (C14), trypsin, α‐chymotrypsin, α‐mannosidase, and α‐fucosidase. Acidification reactions are positive for saccharose, glucose, arabinose, lactose, maltose, raffinose, xylose, trehalose, and rhamnose but negative for glycerol, cellobiose, melezitose, sorbitol, mannitol, and salicin. This strain fails to form indole, is urease negative, and cannot hydrolyze gelatin or esculin. Strain Marseille‐P3236^T^ is able to ferment d‐saccharose, d‐arabinose, d‐xylose, d‐glucose, d‐fructose, d‐ribose, d‐mannose, d‐glucose, l‐rhamnose, erythritol, salicin, methyl‐αd‐mannopyranoside, l‐arabinose, d‐cellobiose, d‐fructose, d‐galactose, d‐raffinose, d‐mannitol, d‐tagatose, d‐sorbitol, d‐maltose, arbutin, d‐lactose, methyl‐αd‐glucosamine, amygdaline, *n*‐acetylglucosamine, esculin ferric citrate, d‐trehalose, d‐melibiose, inulin, d‐melezitose, d‐turanose, starch, and potassium gluconate but not xylitol, potassium 5‐ketogluconate, potassium 2‐ketogluconate, methyl‐βd‐xylopyranoside, l‐xylose, l‐sorbose, l‐fucose, l‐arabitol, inositol, glycerol, glycogen, d‐ulcitol, d‐lyxose, d‐fucose, d‐arabitol, and d‐adonitol. Its major fatty acid is 12‐methyl‐tetradecanoic acid (46%).

Strain Marseille‐P3236^T^ genome is 6,483,434 bp long with 43.41 mol% of G+C content. The 16S rRNA and genome sequences of *P. timonensis* were deposited in EMBL‐EBI under accession number LT598573 and FQSE01000000, respectively [32]. The type strain is Marseille‐P3236^T^ (=CSUR P3236 = CCUG 71183), and was isolated from the stool sample of a healthy 39‐year‐old pygmy male from Congo.

## CONFLICT OF INTEREST

The authors declare no conflict of interest.

## Data Availability

16S rRNA gene sequence was deposited under the accession number: LT598573. The genome bioproject was deposited under the accession number: PRJEB18032. The strain was deposited under the following strain deposit numbers: CSUR P3236 and CCUG 71183.
